# The transposed-word effect provides no unequivocal evidence for parallel processing

**DOI:** 10.3758/s13414-023-02721-5

**Published:** 2023-05-15

**Authors:** Sara V. Milledge, Neya Bhatia, Loren Mensah-Mcleod, Pallvi Raghvani, Victoria A. McGowan, Mahmoud M. Elsherif, Michael G. Cutter, Jingxin Wang, Zhiwei Liu, Kevin B. Paterson

**Affiliations:** 1 School of Psychology and Computer Science, University of Central Lancashire, Preston, UK; 2 School of Psychology and Vision Sciences, University of Leicester, Leicester, UK; 3 Department of Psychology, University of Birmingham, Birmingham, UK; 4 School of Psychology, University of Nottingham, Nottinham, UK; 5 Academy of Psychology and Behavior, Faculty of Psychology, Tianjin Normal University, Tianjin, China; 6 School of Education and Psychology, Sichuan University of Science and Engineering, Zigong, China

**Keywords:** Reading, Parallel processing, Serial processing, Transposed-word effect

## Abstract

Studies using a grammaticality decision task have revealed surprising flexibility in the processing of word order during sentence reading in both alphabetic and non-alphabetic scripts. Participants in these studies typically exhibit a transposed-word effect, in which they make more errors and slower correct responses for stimuli that contain a word transposition and are derived from grammatical as compared to ungrammatical base sentences. Some researchers have used this finding to argue that words are encoded in parallel during reading, such that multiple words can be processed simultaneously and might be recognised out of order. This contrasts with an alternative account of the reading process, which argues that words must be encoded serially, one at a time. We examined, in English, whether the transposed-word effect provides evidence for a parallel-processing account, employing the same grammaticality decision task used in previous research and display procedures that either allowed for parallel word encoding or permitted only the serial encoding of words. Our results replicate and extend recent findings by showing that relative word order can be processed flexibly even when parallel processing is not possible (i.e., within displays requiring serial word encoding). Accordingly, while the present findings provide further evidence for flexibility in the processing of relative word order during reading, they add to converging evidence that the transposed-word effect does not provide unequivocal evidence for a parallel-processing account of reading. We consider how the present findings may be accounted for by both serial and parallel accounts of word recognition in reading.

## Introduction

Within reading research, there is a longstanding debate concerning whether words are recognised serially or in parallel. Serial-processing accounts stipulate that attention is allocated to one word at a time and that words are recognised in the order that the reader encounters them (e.g., E-Z Reader model – Reichle et al., 1998, 2003). By contrast, parallel-processing accounts propose that multiple words are encoded simultaneously, within a narrow spatial region around the readers’ current point of fixation (e.g., SWIFT – Engbert et al., 2005; Glenmore, Reilly & Radach, 2006; OB1-Reader – Snell et al., 2018).

Within serial accounts, it is argued that parallel encoding is implausible as it would limit the reader’s ability to keep track of word order (Reichle et al., 2009; White et al., 2019). However, within parallel-processing accounts it is argued that such problems are inherent to reading (Snell & Grainger, 2019b), based on studies showing that readers will sometimes misprocess word order (Mirault et al., 2018; Snell & Grainger, 2019a; see also Kennedy & Pynte, 2008). Relevant recent evidence for this comes from experiments in which participants made speeded grammaticality decisions for short (typically five-word) sentences in which two adjacent words were transposed. These transposed-word stimuli were created from base forms of the sentences that were either grammatical (e.g., “The white cat was big” becomes “The white was cat big” following word transposition) or ungrammatical (e.g., “The white cat was slowly” becomes “The white was cat slowly” following word transposition), with word transpositions always producing an ungrammatical sentence. Intermixed with these stimuli, the experiments typically include an equal number of grammatical sentence stimuli, constructed similarly to the experimental stimuli (i.e., five words in length).

The key findings from these experiments is that participants make more errors and are slower to respond correctly to transposed-word stimuli derived from grammatical than ungrammatical base sentences. This is taken as evidence for flexible word-order processing. Specifically, it is argued that uncertainty in the relative spatial encoding of words allows participants to access a mental representation of the base forms of stimuli derived from grammatical sentences (i.e., with words in the correct order), interfering with their ability to correctly categorise these stimuli as ungrammatical. By comparison, transposed-word stimuli derived from ungrammatical base sentences should not create such interference, and so should be easier to categorise as ungrammatical.

This transposed-word effect has been demonstrated numerous times in French (e.g., Mirault et al., 2018, 2020; Pegado & Grainger, 2020; Snell & Grainger, 2019a; Wen et al., 2022) and more recently in Chinese (Liu et al., 2020, 2021, 2022). Within a parallel-processing account (e.g., OB1-Reader; Snell et al., 2018), the effect can be explained in terms of the noisy (i.e., imprecise) mapping of relative word locations during reading (Snell & Grainger, 2019a, b). Within such models, multiple words can be active and recognised in parallel, although the system may have difficulty keeping track of their order, as the spatiotopic representation of sentence structure that the (multiple) words are mapped onto in short-term memory is imprecise. This imprecision in mapping is thought to allow top-down syntactic and contextual knowledge to influence word-order processing. As such, sentences with an ungrammatical word order may be ‘corrected’ by expectation-driven processing, leading to ungrammatical sentences being assigned a plausible interpretation.

Other accounts have argued that parallel word encoding is not required to produce such effects, and that words could be processed serially and then subsequently mentally re-ordered. For example, noisy-channel models of sentence processing allow for the syntactic processor to edit or re-order elements of its input based on semantic knowledge, to correct for errors in the input and to enable a plausible interpretation of meaning to be achieved (e.g., Gibson et al., 2013). Crucially, such models make no claims as to whether word encoding is serial or parallel, but allow for word order to be revised at a post-lexical stage of processing. Consequently, while it is argued that flexible word-order processing can be explained via parallel word encoding (Snell & Grainger, 2019b), this flexible processing also can be achieved within a model in which words are encoded serially (see Huang & Staub, 2021a, b). This raises the possibility that parallel processing might not be necessary to produce the transposed-word effect. This was tested recently by Liu et al. (2022) in Chinese, using a speeded grammaticality decision task and text presentation procedures that either allowed for parallel word encoding (i.e., sentence stimuli were displayed normally, e.g., Mirault et al., 2018) or required that words were encoded serially (i.e., by presenting the words in each sentence stimulus sequentially at a central screen location). Crucially, Liu et al. observed a transposed-word effect using both presentation procedures (although the effect was smaller and observed only in error rates for serial word presentations), which they took as evidence that the transposed-word effect does not depend on parallel word encoding.

As these findings were reported in Chinese and the transposed-word effect was originally demonstrated using an alphabetic script (French), it will be important to establish whether Liu et al.’s (2022) findings can be replicated in other scripts. Moreover, such an approach is consistent with the increasing recognition that replication studies are crucial for confirming (or disconfirming) important findings and establishing boundary conditions (e.g., Shrout & Rodgers, 2018). Accordingly, with the present study, we examined whether these findings could be replicated in English. As in previous research, participants provided speeded grammaticality decisions for ungrammatical transposed-word stimuli derived from grammatical and ungrammatical base sentences, intermixed with an equal number of grammatical sentences. These stimuli were presented in three experiments using procedures that either allowed or disallowed parallel word encoding. To permit parallel encoding, stimuli were presented as whole sentences, with all their constituent words visible simultaneously (Experiment 1). To ensure serial encoding, the words in each stimulus were presented sequentially either at a central screen location (Experiment 2) or progressively across the screen at the same spatial locations they would occupy within a standard sentence presentation (Experiment 3). Experiments 1 and 2 therefore used the same stimulus presentation procedures as Liu et al., while Experiment 3 used a novel presentation procedure that enabled us to go beyond this previous work by assessing whether, and how, displaying words serially while maintaining the spatial location of a given word within a sentence might influence the transposed-word effect.

## Method

### Participants

In total, 197 participants, aged 18–33 years old, were recruited across the three experiments (Experiment 1, 64 participants, 49 female; Experiment 2, 67 participants, 55 female; Experiment 3, 66 participants, 50 female). Different participants took part in each experiment. Sample sizes for each experiment were similar to those in previous research reporting a transposed-word effect (e.g., Mirault et al., 2018, Experiment 1), and exceeded the minimum of 1,600 observations per condition recommended by Brysbaert and Stevens (2018) for within-subject designs using reaction time (RT) measures.

Participants were recruited from the School of Psychology at the University of Leicester and via Prolific (a platform for recruiting participants for behavioural studies online). Participants from the School of Psychology were awarded course credits for their participation; participants recruited via Prolific were paid at a standard rate of £10/h for their participation. Using Prolific, four participants were recruited for Experiment 1, 20 were recruited for Experiment 2, and 16 were recruited for Experiment 3. Participants recruited via Prolific were matched to the University of Leicester participants with regard to age range (18–33 years old), language (all participants had to be native English speakers), geographical location (participants had to live within the UK), and education (participants had to be completing an undergraduate degree). All participants were required to have normal or corrected-to-normal vision and to have no known language or reading difficulties. The research was approved by the School of Psychology Research Ethics Committee at the University of Leicester and conducted in accordance with the principles of the Declaration of Helsinki.

### Stimuli and design

Stimuli were constructed using the same procedure as Mirault et al. (2018). Each experiment used 200 five-word sentences as stimuli: 50 were experimental stimuli created by transposing two adjacent words from a grammatical base sequence to create an ungrammatical sentence (TW condition); 50 were control stimuli created by transposing two adjacent words in an ungrammatical base sequence to create a sentence that was still ungrammatical (Control condition); and 100 were grammatically correct sentences (Grammatical condition), which were randomised and intermixed with the ungrammatical sentences for the purpose of the grammaticality decision task (see Table 1 for an example of how the sentences were constructed).

**Table 1:** Example of how Control and Transposed word (TW) conditions were created

Base sequence	Sentences
Grammatical	The dirty clothes were disgusting
	The large ship sailed slowly
Ungrammatical	The dirty clothes were slowly
	The large ship sailed disgusting
Test sequence	
TW	The dirty were clothes disgusting
	The large sailed ship slowly
Control	The dirty were clothes slowly
	The large sailed ship disgusting

Though the same set of stimuli were used in each experiment, the display procedures differed between the experiments. In Experiment 1, whole sentences were presented on the screen until participants pressed a response key. In Experiment 2, the sentences were presented one word at a time at a fixed central location for a specified period of 250 ms each. In Experiment 3, the sentences were presented one word at a time (again, for a fixed period of 250 ms per word), moving progressively across the screen so that the words appeared in the same spatial location as they would within a full sentence presented normally.

We examined the effects of condition (TW, Control) and display procedure on error rates and latencies of correct responses. In Experiment 1, the response time (in ms) was measured from the onset of the sentence until a response key was pressed. In Experiments 2 and 3, this was measured from when the last word in the sentence appeared on the screen.

### Apparatus and procedure

The experiments were conducted using Gorilla.sc, a browser-based platform for the remote collection of behavioural research data (Anwyl-Irvine et al., 2020), which has been shown to provide high precision measurement of RTs even with non-optimal setups (Bridges et al., 2020).

Stimuli were presented via participants’ desktop or laptop computers and responses were recorded via participants’ keyboards, using the ‘J’ and ‘K’ keys for grammatical and ungrammatical decisions, respectively.

The procedure was explained to participants at the start of each experiment. Participants were instructed to use the ‘J’ and ‘K’ keys to indicate as quickly and as accurately as possible whether each sentence was grammatically correct. Participants were also instructed to activate a full-screen mode, to ensure standardised presentation of the stimuli. Each trial began with a fixation cross, presented for 250 ms. This was then replaced by a stimulus display. Six practice trials were included to familiarise participants with the task and procedure.

**Fig. 1:**
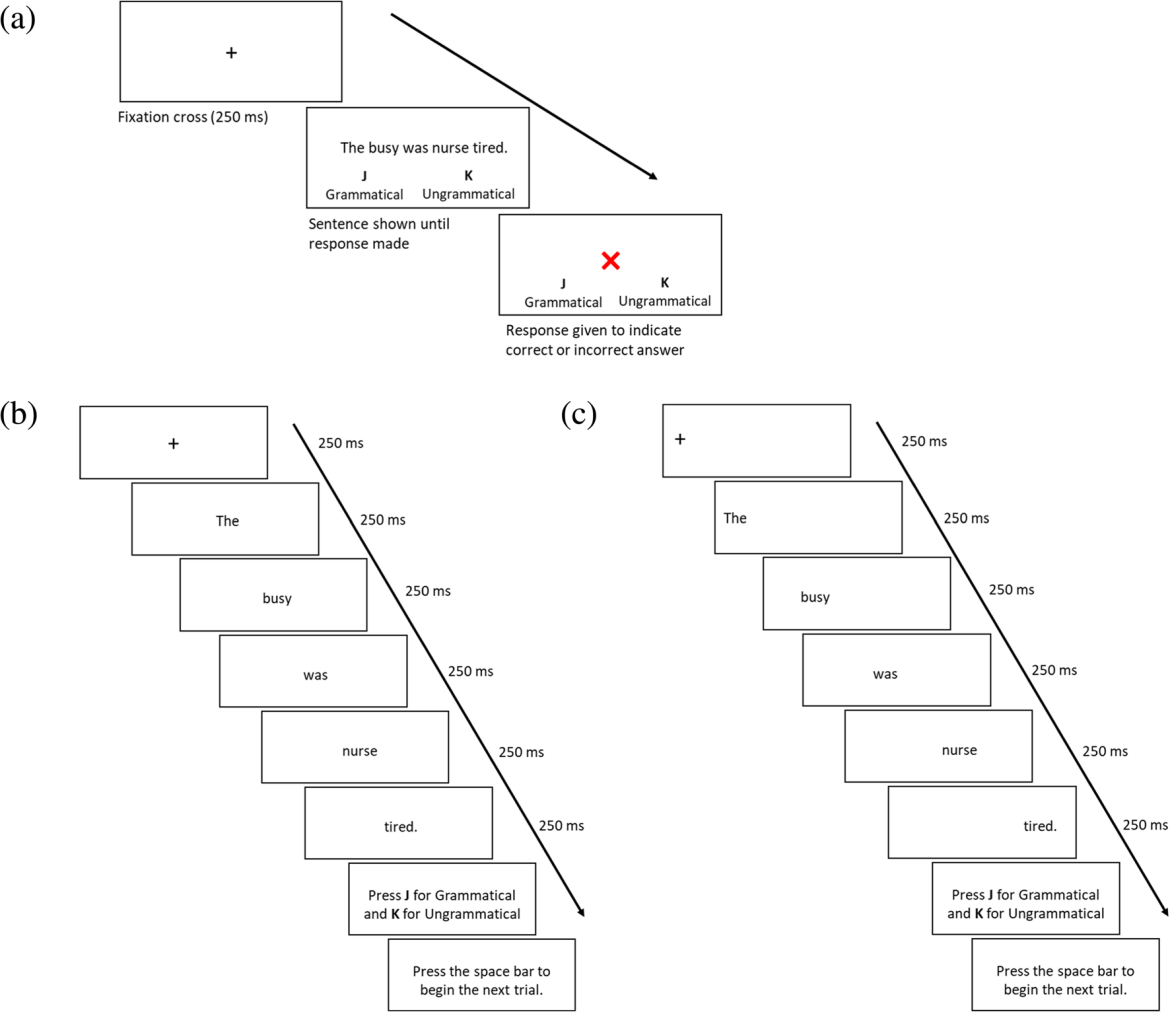
Example trial procedures for Experiments: (**a**) Experiment 1 used full sentence presentations, (**b**) Experiment 2 used sequential, centralised word-by-word presentations, and (**c**) Experiment 3 used sequential, progressive word-by-word presentations

In Experiment 1, each sentence stimulus was presented on the screen in full until participants pressed a response key (or the display timed out). The next trial automatically began after this screen. In Experiment 2, each sentence stimulus was presented one word at a time at a fixed central location for 250 ms. In Experiment 3, each sentence stimulus was presented one word at a time (for 250 ms), but, unlike in Experiment 2, the words were presented progressively across the screen so that each word appeared in the same location as it would within the sentence. In both experiments, participants made a response following the final word in the sequence. In Experiments 2 and 3, after a grammaticality decision was made, participants used the space bar to move on to the next trial. After each trial in all three experiments, either a green tick or a red cross was shown as feedback for correct and incorrect responses, respectively. Each experiment lasted approximately 15 min per participant. Figure 1 illustrates the trial procedure for each experiment.

## Results

Performance for the grammatically correct (Grammatical) stimuli was used to assess whether participants were engaging with the task, using predefined exclusion criteria of less than 80% response accuracy and latencies of responses that exceeded 2.5 SDs from the mean. With regard to response accuracy, this led to four participants being excluded from Experiment 1 (leaving 60 participants), five from Experiment 2 (leaving 62 participants), and seven from Experiment 3 (leaving 59 participants). Applying the exclusion procedure for RTs to the remaining data removed 3.52% of data for Experiment 1, 0.94% for Experiment 2, and 2.42% for Experiment 3. The final sample sizes following the application of these exclusion criteria were in line with other transposed-word experiments (e.g., Mirault et al., 2018), while the number of observations per condition (Experiment 1 = 3,000, Experiment 2 = 3,100, Experiment 3 = 2,950) remained larger than the minimum recommended by Brysbaert and Stevens (2018). Error rates and the latencies of correct responses were analysed for each experiment, comparing the Control and TW conditions (see Table 2; the Grammatical condition is included in the table for comparison).

**Table 2:** Means and standard deviations (in parentheses) for error rates and latencies of correct responses for Experiments 1, 2 and 3

	Control	TW	Grammatical
	Experiment 1		
Error rate (%)	5.37 (23)	12.49 (33)	3.48 (18)
Reaction time (ms)	1625.09 (579)	1651.36 (590)	1382.20 (524)
	Experiment 2		
	Control	TW	Grammatical
Error rate (%)	4.48 (21)	14.19 (35)	4.32 (20)
Reaction time (ms)	631.43 (506)	637.12 (536)	581.91 (460)
	Experiment 3		
	Control	TW	Grammatical
Error rate (%)	5.15 (22)	11.83 (32)	3.88 (19)
Reaction time (ms)	511.94 (364)	533.74 (388)	504.30 (353)

**Fig. 2:**
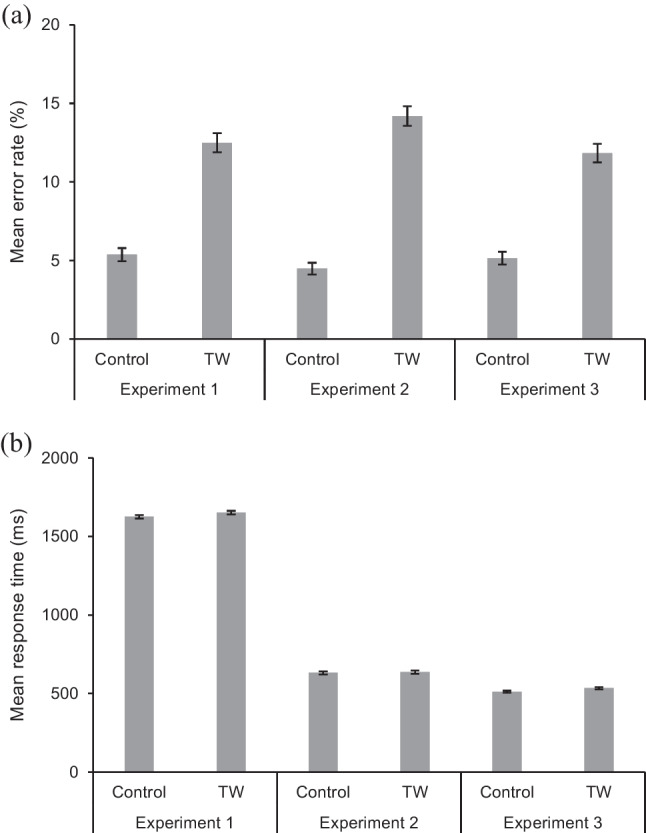
(**a**) Mean error rates and (**b**) mean latencies of correct responses for experiments. *Note.* Error bars represent standard error

Data were analysed in generalized linear mixed effects (GLME) models (Lo & Andrews, 2015), using the *glmer* function from the lme4 package (Bates et al., 2015) within the R environment for statistical computing (R Core Team, 2020). Participants and items were entered as crossed random effects. For each experiment, two models were run (one for error rates, one for latencies of correct responses) to compare the two key conditions (Control, TW). We first report analyses for each experiment separately. We then report two further models that combined data from the experiments and included the variable Experiment as an interactive term to compare effects across the different display procedures. The *contr.sdif* function (package MASS) was used to set up factors. Following convention, effects were considered significant when |*z*| or |*t*| > 1.96, with *p*-values calculated using the *lmerTest* package. Table 2 shows mean error rates and response latencies for correct responses, Table 3 summarises the statistical effects for each experiment, Table 4 summarises statistical effects for the combined data, and Fig. 2 illustrates the mean data.

**Table 3:** Summary of statistical effects for each experiment

Effect	Error rates				Latencies of correct responses			
	b	SE	*z*	*p*	b	SE	*t*	*p*
*Experiment 1*								
Intercept	-2.88	.15	-19.83	< .001	1688.08	4.18	404.19	< .001
TW vs. Control	1.10	.15	7.27	< .001*	19.81	5.19	3.82	< .001*
*Experiment 2*								
Intercept	-2.84	.14	-19.77	< .001	654.06	5.01	130.54	< .001
TW vs. Control	1.27	.16	8.00	< .001*	14.95	4.64	3.23	.001*
*Experiment 3*								
Intercept	-3.23	.20	-16.56	< .001	535.44	12.00	44.61	< .001
TW vs. Control	1.43	.19	7.57	< .001*	26.33	10.16	2.59	.010*

Significant effects are indicated using an asterisk

**Table 4:** Summary of statistical effects for combined analyses

Effect	Error rates				Latencies of correct responses			
	b	SE	*z*	*p*	b	SE	*t*	*p*
Intercept	-2.95	.10	-30.91	< .001	949.36	2.38	399.22	< .001
TW vs. Control	1.27	.10	13.13	< .001*	21.46	2.27	9.45	< .001*
Exp. 1 vs. 2	-.08	.21	-.39	.699	-980.90	2.01	-487.79	< .001*
Exp. 2 vs. 3	-.23	.21	-1.08	.282	-123.85	1.92	-64.50	< .001*
TW vs. Control × Exp. 1 vs. 2	.31	.20	1.57	.117	-8.93	1.81	-4.94	< .001*
TW vs. Control × Exp. 2 vs. 3	-.15	.21	-.72	.457	6.45	1.79	3.61	< .001*

Significant effects are indicated using an asterisk

### Experiment 1: Whole sentence presentations

#### Error rates

Participants made significantly more errors in the TW condition compared to the Control condition.

#### Latencies of correct responses

Participants were slower to respond in the TW condition compared to the Control condition.

### Experiment 2: Central serial presentations

#### Error rates

Participants made significantly more errors in the TW condition compared to the Control condition.

#### Latencies of correct responses

Participants were slower to respond in the TW condition compared to the Control condition.

### Experiment 3: Progressive serial presentations

#### Error rates

Participants made significantly more errors in the TW condition compared to the Control condition.

#### Latencies of correct responses

Participants were slower to respond in the TW condition compared to the Control condition.

### Combined analyses of experiments 1–3

#### Error rates

Participants made significantly more errors in the TW condition compared to the Control condition. The absence of significant interactions suggests that this pattern was consistent across all three experiments.

#### Latencies of correct responses

Participants were slower to respond in the TW condition compared to the Control condition. There were also significant differences in latencies of correct responses between the experiments; RTs were faster in Experiment 2 compared to Experiment 1, and faster in Experiment 3 compared to Experiment 2. Although numerical differences show that across all three experiments participants displayed longer RTs in the TW condition compared to the Control condition, the magnitude of this effect was smaller in Experiment 2 than Experiment 1, and larger in Experiment 3 than Experiment 2 (reflected by the significant interaction terms for “TW vs. Control × Exp. 1 vs. 2” and “TW vs. Control × Exp. 2 vs. 3”, respectively).

### Bayes factors for between-experiment interaction effects

The mixed-effect GLMEs we conducted to compare errors for TW vs. Control stimuli across experiments produced null effects. These analyses do not allow us to draw firm conclusions, as it is uncertain whether the null effect represents the absence of an effect or the failure to detect an effect that is genuinely present in the data. Accordingly, to explore this issue further, we conducted additional analyses using a Bayesian approach (see, e.g., Dienes, 2019; Kass & Raftery, 1995; Morey et al., 2016) to quantify statistical evidence for the null hypothesis (i.e., that error rates were indeed consistent across experiments). Bayes factors (*BF*s) were calculated from the interaction estimates produced by the GLMEs, using the motivated maximum approach (Silvey et al., 2021; see Dienes, 2014). Given limited prior evidence for a difference in error rates for TW versus Control stimuli across the stimulus presentation procedures we employed, we used a normal distribution rather than a half normal distribution to calculate *BF*s. These quantified the statistical evidence for the presence of an interaction (*H*_1)_ versus its absence (*H*_0_). Following the approach proposed by Lee and Wagenmakers (2014; based on Jeffreys, 1939), we considered *BF*_10_ > 3 to constitute ‘moderate’ evidence for *H*_1_, and *BF*_10_ < .33 to constitute ‘moderate’ evidence for *H*_0_, with values between these levels providing equivocal evidence. We conducted separate analyses comparing errors for TW compared to Control stimuli in Experiment 1 versus 2, and the same comparison for Experiment 2 versus 3. The resulting *BF*s were smaller than .33 (TW vs. Control × Exp. 1 vs. 2: BF_N(0,.20)_ = .23; TW vs. Control × Exp. 2 vs. 3: BF_N(0,.21)_ = .09), indicating that we have at least moderate evidence for the null hypothesis in both cases. From these analyses, we can infer that error rates for TW versus Control stimuli were similar across the different stimulus presentation procedures used in the experiments.

## Discussion

With the present study, we investigated whether the transposed-word effect depends on the parallel encoding of words during reading. Following Liu et al. (2022), we used a speeded grammaticality decision task and stimulus presentation procedures that either allowed for parallel word encoding (Experiment 1) or required that words were encoded serially (Experiments 2 and 3). Consistent with Liu et al.’s findings from Chinese, we observed a transposed-word effect in grammaticality decision errors for both types of stimulus presentation. This provides further evidence, this time from English, that a transposed-word effect can be obtained even when words must be processed serially. Accordingly, while our results show that relative word order can be processed flexibly (e.g., Mirault et al., 2018), they present a further challenge to the claim that such effects depend, crucially, on words being recognised in parallel (e.g., Snell & Grainger, 2019a, b).

In our view, this finding provides strong evidence against a parallel-processing account of the transposed-effect, as the effect can be observed even when words must be encoded serially. It nevertheless was of concern to establish whether the effect differs across presentation conditions, as this might provide further insights into the underlying mechanisms. Liu et al. (2022) had reported that the transposed-word effect they obtained in grammaticality decision errors was larger for whole sentence compared to serial word presentations, while a transposed-word effect in response latencies for whole sentences was absent when words were presented serially. Liu et al. suggested that the response time effect might be attributable to task differences; namely, whereas participants could provide a grammaticality decision at any point during a whole sentence presentation, for serial word presentations they could only do so after the display of the final word in a sentence. Liu et al. also acknowledged that the difference in the size of the effect in decision errors might reflect a benefit for viewing multiple words simultaneously, and that this could be interpreted by parallel accounts as evidence for the parallel encoding of words. They noted, however, that the effect might be explained within serial accounts in terms of the rapid sequential processing that takes place when reading sentences naturally (e.g., Cutter et al., 2015).

The present study provided less clear evidence for differences in the transposed-word effects for whole sentence compared to serial word presentations, possibly because we employed a between-participants manipulation of stimulus presentation methods. Unlike Liu et al. (2022), we observed essentially the same transposed-word effect in errors for whole sentence and serial word presentations (as confirmed by Bayes factors analyses). However, a larger transposed-word effect in response times for whole sentences (Experiment 1) compared to when words were presented serially at a central screen location (Experiment 2), was more in line with Liu et al.’s findings. More intriguingly, the RT effect was also larger for progressive serial presentations (Experiment 3) compared with centrally displayed serial word presentations. Such comparisons are potentially confounded by the same procedural differences for whole sentence versus serial word presentations as in Liu et al.’s study. However, they may provide some further indication of a stronger transposed-word effect for whole sentence compared to (centrally-presented) serial word presentations. Moreover, the stronger effect for progressive versus central serial word presentations raises the possibility that positional uncertainty may be greater when the relative spatial locations of words is preserved. We note that preserving the relative spatial locations of words provides a more naturalistic presentation technique compared to presenting words centrally, which could have contributed to this effect (and for a similar discussion, see Dufour et al., 2022).

Interestingly, two other recent follow-ups to the Liu et al. (2022) study have also looked more closely at differences in the transposed-word effects for whole sentence compared with serial word presentations (Huang & Staub, 2022; Mirault et al., 2022). Both studies addressed the potential procedural confound in the original Liu et al. study by assessing grammaticality decisions for serial word presentations during which participants could respond either at any point during a sentence presentation or only following its final word. Huang and Staub focused on error rates, using stimuli presented in English. Like Liu e al., they obtained a larger transposed-word effect for whole sentence as compared with both types of serial word presentation (which did not differ from each other). Likewise, but using stimuli in French, Mirault et al. obtained a larger transposed-word effect for whole sentences compared with both types of serial word presentation. Mirault et al. also reported a transposed-word effect in response times for whole sentence presentations that was absent for serial word presentations, replicating Liu et al.’s findings. Accordingly, with the possible exception of the present study, current investigations suggest that the transposed-word effect is larger or more robust for whole sentence compared to serial word presentations, potentially because flexibility in word-order processing is better supported when viewing multiple words at a time during reading.

As noted above, such findings might be interpreted as evidence for a stronger transposed-word effect when parallel word encoding is possible. For example, Mirault et al. (2022) argue that transposed-word effects are weaker when serial reading is imposed precisely because this reduces bottom-up uncertainty about relative word locations. They propose that transposed-word errors will be observed, to a lesser extent, when words in sentences must be processed serially as a consequence of ‘good-enough’ processing aimed at obtaining a plausible interpretation of the linguistic input (e.g., Ferreira & Lowder, 2016; see also Gibson et al., 2013). This is assumed to be achieved though a combination of less noisy bottom-up information about relative word order and top-down contraints imposed by syntactic and contextual expectations, as specified within the OB1-Reader model (Snell et al., 2018; see also Wen et al., 2021). Note that this account would also predict a stronger word-transposition effect when words are presented serially but occupy their normal spatial locations in sentences (as in the present Experiment 3), as this would preserve the uncertainty about relative word locations obtained for standard sentence presentations. Crucially, however, such an account implicitly acknowledges that the transposed-word effect does not provide unequivocal evidence for a parallel-processing account, as it recognizes that the effect can be observed in grammaticality decision errors even under serial reading conditions.

Critically, serial processing accounts also assume that readers can benefit from viewing multiple words simultaneously (e.g., by facilitating the parafoveal pre-processing of the next word in a sentence; see Liu et al., 2022, for a discussion) without invoking parallel word encoding. For instance, inspired by noisy channel models of sentence processing (e.g., Gibson et al., 2013), Huang and Staub (2021a, 2021b) have argued for a serial processing account in which words are recognized sequentially during reading, but where their subsequent integration within a memory representation of the sentence meaning is not strictly serial. In particular, Huang and Staub argue that when the word that currently is being processed cannot be integrated easily within this memory representation, the integration decision may be deferred until the next word (or words) is processed, so that the reader might potentially hold two (or more) words temporarily in an unintegrated state. Crucially, this allows for the possibility of the next word in a sentence being integrated ahead of the first word if it better fits with the sentence context, providing a mechanism for readers to ‘correct’ word order errors during post-lexical processing. Huang and Staub (2022) additionally propose that readers are more likely to attribute erroneous word sequences in whole-sentence presentations (as compared to serial word presentations) to an eye movement error causing words to be processed in the wrong order (see also Staub et al., 2019). They argue that, in such a situation, it may even be more likely that post-lexical processing will attempt to correct for an apparent error in the input by integrating words in an order that enables the reader to obtain a plausible interpretation of sentence meaning. This might also provide a serial-based explanation for why the transposed-word effect is stronger for progressive versus central serial word presentations in the present study, as normal eye movement behavior is preserved under progressive, but not central, word presentation conditions. Consequently, readers might also be more likely to attribute apparent word order errors in progressive serial word presentations to eye movement error.

Accordingly, while the transposed-word effect has been argued to provide evidence for parallel processing during reading, it should be clear that this effect is not incompatible with a serial processing account. Indeed, the present research, along with other recent studies (Huang & Staub, 2022; Liu et al., 2022; Mirault et al., 2022), provides a compelling demonstration that relative word order can be processed flexibly even when words must be encoded serially. The present findings, therefore, add to converging evidence that parallel processing is not required to support flexible word-order processing, although the underlying mechanisms and potential benefits of viewing multiple words simultaneously remain to be more fully understood. Crucially, for researchers to better understand how word order is processed moment-to-moment during reading, further research, using techniques like eye-tracking (e.g., Huang & Staub, 2020, 2021a), is likely to be needed.
